# Structure–Function Analysis of the Biotechnologically Important Cytochrome P450 107 (CYP107) Enzyme Family

**DOI:** 10.3390/biom13121733

**Published:** 2023-12-01

**Authors:** Tiara Padayachee, David C. Lamb, David R. Nelson, Khajamohiddin Syed

**Affiliations:** 1 Department of Biochemistry and Microbiology, Faculty of Science, Agriculture and Engineering, University of Zululand, KwaDlangezwa 3886, South Africa; teez07padayachee@gmail.com; 2 Faculty of Medicine, Health and Life Sciences, Swansea University, Swansea SA2 8PP, UK; d.c.lamb@swansea.ac.uk; 3 Department of Microbiology, Immunology and Biochemistry, University of Tennessee Health Science Center, Memphis, TN 38163, USA; drnelson1@gmail.com

**Keywords:** P450, CYP107, crystal structure, active site, enzymatic reaction, substrate, secondary metabolites, amino acid dynamics, polar and hydrophobic interactions

## Abstract

Cytochrome P450 monooxygenases (CYPs; P450s) are a superfamily of heme-containing enzymes that are recognized for their vast substrate range and oxidative multifunctionality. CYP107 family members perform hydroxylation and epoxidation processes, producing a variety of biotechnologically useful secondary metabolites. Despite their biotechnological importance, a thorough examination of CYP107 protein structures regarding active site cavity dynamics and key amino acids interacting with bound ligands has yet to be undertaken. To address this research knowledge gap, 44 CYP107 crystal structures were investigated in this study. We demonstrate that the CYP107 active site cavity is very flexible, with ligand binding reducing the volume of the active site in some situations and increasing volume size in other instances. Polar interactions between the substrate and active site residues result in crucial salt bridges and the formation of proton shuttling pathways. Hydrophobic interactions, however, anchor the substrate within the active site. The amino acid residues within the binding pocket influence substrate orientation and anchoring, determining the position of the hydroxylation site and hence direct CYP107’s catalytic activity. Additionally, the amino acid dynamics within and around the binding pocket determine CYP107’s multifunctionality. This study serves as a reference for understanding the structure–function analysis of CYP107 family members precisely and the structure–function analysis of P450 enzymes in general. Finally, this work will aid in the genetic engineering of CYP107 enzymes to produce novel molecules of biotechnological interest.

## 1. Introduction

Cytochrome P450 monooxygenases (CYPs/P450s) are a superfamily of heme-containing enzymes known for their broad substrate specificities and diverse catalytic activities. Genomic sequence analysis of P450s in *Bacteria* has shown that these enzymes are frequently located in operons and biosynthetic gene clusters (BGCs) [1,2,3,4], functioning as oxidative tailoring enzymes, leading to functional diversity in the generated secondary metabolites [5,6]. Due to their catalytic diversity with stereo- and regio-specific oxidation capabilities, P450 enzymes have been exploited for various applications, including biomedical and biotechnological applications [7,8,9,10,11,12].

Using an established nomenclature procedure, P450 enzymes are classified into different P450 families and subfamilies based upon their percentage amino acid sequence identities. If the shared P450 sequence is more than 40% identical, they belong to the same family, e.g., CYP1A1; if sequences share more than 55% identity, they belong to the same subfamily, e.g., CYP1A1, CYP1A2 [13]. Since their initial identification six decades ago, most P450s that have been identified have been grouped into different P450 families [14]. As of 2023, 1910 P450 families have been identified in *Bacteria* [14]. Among the *Bacteria* P450 families, the CYP107 family is found to be dominantly present in BGCs, such as in *Streptomyces* [15], *Firmicutes* [16], and Gamma- and Delta-*proteobacteria* [2], indicating key role(s) for CYP107 family members in the synthesis of many BGC-derived secondary metabolites. *Streptomyces* species produce two-thirds of all microbial-derived secondary metabolites, including antibiotics [17], and CYP107 is dominant in their BGCs [15].

CYP107 family members perform hydroxylation and epoxidation reactions in a wide range of substrates, as summarized in Table 1. CYP107 family members can function as oxidative tailoring enzymes in macrolide antibiotic biosynthetic pathways, such as those generating erythromycin and mycinamicin [18,19]. In *Streptomyces* species, CYP107 family members are involved in the biosynthesis of many valuable human antibiotics, including pikromycin and rapamycin [20,21]. CYP107H1 (P450BioI), found in *Bacillus subtilis*, synthesizes pimelic acid, a major component of biotin (vitamin B7) [22]. This P450 catalyzes oxidative cleavage at the C7-C8 position of ACP-linked fatty acids (Table 1) [22]. CYP107BR1 (P450vdh) found in *Pseudonocardia autotrophica* biosynthesizes vitamin D3 by performing a two-step hydroxylation (Table 1) [23]. Due to their potential biotechnological value, many CYP107 proteins have been functionally characterized, and their role in synthesizing various secondary metabolites has been elucidated (Table 1).

The CYP107 members studied to-date are soluble proteins, and their general overall structure is comparable to that of bacterial P450s [39,40]. Structural analysis of CYP107 members revealed characteristic P450 folds and conserved motifs in their structures [39,40]. The α/β structure of CYP107 members comprises two structural domains: a helical domain and random coils and β-sheets [18,22,41]. The conserved catalytic residues and characteristic P450 structure, which includes a kink in the I-helix where the highly conserved acid/alcohol residue pair controls the protonation of intermediate oxygen species during oxygen activation, are present in all CYP107 members’ active sites, except for CYP107A1 [18,26]. The proximal cysteine heme thiolate ligand is located in the *N*-terminal loop before the L-helix. It is often exposed to solvents via the substrate-binding pocket, which is covered by three domains, namely the F1, F2, and F3 loops [21,22] The active site contains various hydrophobic regions interacting with the substrate [22,35]. CYP107 members generally require endogenous ferredoxin and ferredoxin reductase partner proteins to perform their catalytic functions. Functional analysis of some CYP107 members in vitro revealed that exogenous ferredoxin and NADPH-ferredoxin reductase from spinach and reductase domain (RhFRED) from *Rhodococcus* in partnership with spinach ferredoxin can transfer the electrons [19,27,42].

Over the past few years, the resolved X-ray crystal structures of many CYP107 family members’ have been determined. To-date, 44 CYP107 family members’ crystal structures have been deposited and are available for use at the Research Collaboratory for Structural Bioinformatics (RCSB) Protein Data Bank (PDB) [43]. From the above data (Table 1), the structure–function analysis of individual CYP107 enzymes has been well established. However, these studies focus on CYP107 enzymes in isolation, oxidizing a specific substrate. A comprehensive comparative structure–function analysis of CYP107 family members, including amino acid dynamics, and their defined role(s) in catalysis has yet to be reported. The availability of 44 CYP107 family protein crystal structures allows us to decipher the detailed structure–function mechanics of these P450s, with a particular focus on amino acid dynamics. Herein, this study is aimed at addressing this research knowledge gap. We have analyzed 44 crystal structures and delineated the structure–function relationships between CYP107 family members.

## 2. Materials and Methods

### 2.1. Retrieving of CYP107 Member’s Structures

CYP107-member protein crystal structures were retrieved from RCSB PDB [43] and used in this study (Table 2). Of the 44 crystal structures, 3 of them, namely CYPTaml, P450Revl, and P450-sb21, had not been previously assigned classical P450 family and subfamily names (Table 2). Assigning the families and subfamilies for these P450s is reported here following the International P450 Nomenclature Committee rules [13].

### 2.2. CYP107 Active Site Analysis

Individual CYP107 crystal structures were analyzed and assigned to either an open (non-ligand bound) or closed (ligand-bound) conformation. Each open and closed CYP107 crystal structure active site area and volume was analyzed by the Computed Atlas of Surface Topography of proteins (CASTp) [44]. For active site analysis, each PDB file was individually uploaded onto PyMOL software, Version 2.2.5 [45]. The active site cavities were selected using heme as the central point of the binding pocket, and amino acid residues within 5 Å were chosen. In cases where the ligand extended out of the selected binding pocket, 5 Å from the ligand was chosen instead. The amino acid residues were analyzed for both open and closed conformations. Active site amino acid dynamics were analyzed by choosing one PDB representative in the open and closed conformation for each CYP107 protein, if available. The amino acid residues and count for each PDB were then compared and analyzed for any changes in the amino acid composition of the active sites. Amino acid residues were represented as sticks and labelled using the three-letter amino acid codes.

### 2.3. Analysis of Ligand Interactions in Closed Conformation PDB Files Using PyMOL

Of the 44 crystal structures, 28 were in the closed conformation. Individual PDB files were uploaded onto PyMOL and the active site cavity was selected, as described above. The amino acids were represented as sticks and labelled according to their single letter amino acid code. Polar contacts with atoms were selected; if the ligand interactions with amino acid residues were present, dashed lines connect the ligand and the specific amino acid residue, water molecule, or solvent molecule. Using published data, the literature, hydrophobic residues within 5 Å were selected and represented as sticks. The amino acid residues that were not interacting with the ligand were removed.

## 3. Results

### 3.1. Enzyme Conformation Affects the Active Site Morphology in CYP107 Proteins

Most CYP107 crystal structures were in a closed conformation (63.6%) (Table 3). The area of the active site cavity of CYP107 proteins co-crystallized with a bound ligand ranged between 789 Å^2^ (CYP107FH5) and 2070 Å^2^ (CYP107G1), whereas the area of the active site in an open conformation ranged between 950 Å^2^ (CYP107Z14) and 2287 Å^2^ (CYP107G1) (Table 3). A similar pattern was noticed when comparing the volume of both conformations. The volume of the active site in a closed conformation ranged between 455 Å^3^ (CYP107FH5) and 2196 Å^3^ (CYP107G1) compared to 859 Å^3^ (CYP107Z14) and 2637 Å^3^ (CYP107G1) in an open conformation (Table 3). These data indicate the overall flexibility and spaciousness of the CYP107 active site cavity when no ligand is bound, allowing for the possible binding of large substrates. Based on the available crystal structures data, one can estimate that the change in active site area and volume from open to closed conformation is 276 Å^2^ and 494 Å^3^, respectively. In most cases, the area and volume of the active site decreased to bind the ligand, which causes a conformational shift from open to closed. These findings indicate a significant difference between the respective values, showing a dynamic change in the active site cavity when a ligand is bound.

A pattern was observed comparing a specific CYP107 subfamily’s active site cavity size (Table 3). The change in area and volume from open to closed conformation for CYP107FH5 was the highest compared to CYP107G1 and CYP107BR1, indicating that the active site cavity became smaller as its area and volume is decreased on binding ligand. In contrast, CYP107E1 and CYP107L1 have the highest area and volume when the ligand is bound (closed conformation), indicating that substrate binding increased the active site cavity area and volume size (Table 3). The molecular dynamics of amino acids were also flexible from open to closed, and vice versa for these CYP107 subfamilies (Table 4). To better understand these observations, we further quantified the amino acid dynamics using root-mean-square difference (RMSD) between the open and closed conformation of the CYP107 members belonging to the same subfamily (Table 4). RMSD analysis revealed a significant change in amino acid positions in CYP107BR1 and CYP107FH5 compared to CYP107L1, CYP107E1, and CYP107G1 (Table 4). CYP107BR1 and CYP107FH5 had the highest RMSD values of 2.5 Å and 3.0 Å (Table 4). These P450s were bound to elongated substrates, which may have contributed to the shift in the locations of amino acid residues in the open conformation. The RMSD values of CYP107L1, CYP107E1, and CYP107G1 were less than 1 Å, indicating that the amino acid’s position stayed relatively constant (Table 4). Compared to CYP107BR1 and CYP107FH5, the substrates bound to these P450s were not elongated and would not require as much shifting. The amino acids unique to closed or open confirmation for a given CYP107 subfamily are listed in Table 4. The amino acids uniquely present in either open or closed conformation that possibly play a key role in catalytic reaction are highlighted.

The active site cavity dynamics of CYP107 members are comparable with other P450s. In human CYP3A4 crystal structures complexed with ketoconazole and erythromycin, there was a similar significant shift in the active site volume; however, the active site volume expanded rather than decreased, increasing from 1173 Å^3^ to 2017 Å^3^ and 2682 Å^3^, respectively. The displacement of the protein backbone to accommodate such large substrates is the cause of this volume increase [46]. A similar pattern was shown in CYP2A6. The volume increased from 251 Å^3^ to 300 Å^3^ when bound with phenacetin; however, the volume decreased to 243 Å^3^ when bound with methoxsalen [47]. These findings highlight the overall flexibility of P450 active sites and the dynamic changes associated with ligand binding.

### 3.2. CYP107H1: Substrate Shape Change and Expulsion of Water Molecules as per Substrate Size

**Figure 1:**
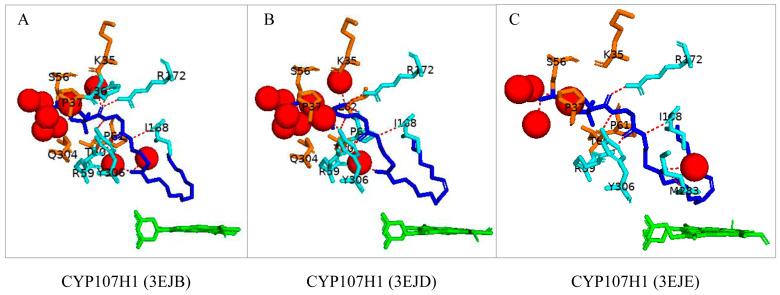
Analysis of CYP107H1 interactions with tetradecanoic acid (**A**), hexadec-9z-enoic acid (**B**), and octadec-9z-enoic acid (**C**). Information on the PDB codes for CYP107H1-fatty acid crystal structures is shown in the figure. Heme is shown in green, and substrates are shown in blue. Amino acid residues sharing a polar interaction with the substrate are shown in cyan, and residues sharing a hydrophobic interaction are shown in orange. Polar interactions are indicated as red dashed lines, water molecules are represented as red spheres, and amino acid residues are labelled according to their one-letter code. A list of all amino acid residues shown in this figure is described in Table 5.

CYP107H1, often referred to as P450bioI, catalyzes the oxidative cleavage of C7–C8 in the acyl carrier protein (ACP)-linked fatty acids involved in pimelic acid synthesis, which makes up the majority of the carbon backbone skeleton of biotin [22]. This P450 interacts with the phosphopantetheine linker of the ACP molecule and forms various hydrogen bonds with active site residues. CYP107H1 has been crystallized with three different chain lengths of fatty acids, namely tetradecanoic acid (Figure 1A), hexadex-9z-enoic acid (Figure 1B), and octadec-9z-enoic acid (Figure 1C). All fatty acids were bound in a U-shaped conformation, bringing the oxidation site closer to the heme Fe [22]. Interestingly, as the fatty acid chain length increased, the number of water molecules interacting with active site residues decreased from 9 to 5, respectively (Figure 1). Met-283 formed a water-mediated hydrogen bond when the largest fatty acid was docked, possibly to provide protons for the oxidation reaction to occur. It has been observed that the larger the fatty acid chain, the closer it was to the heme, and more water molecules were expelled from the binding pocket to increase the space required to bind a larger substrate.

### 3.3. CYP107A1

#### 3.3.1. Perpendicular Binding of the Substrate

**Figure 2:**
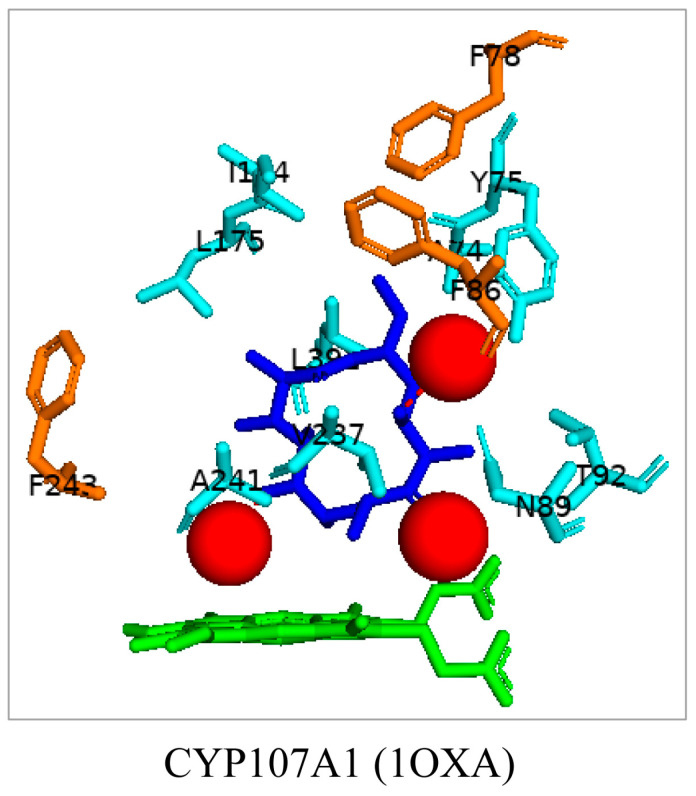
Analysis of CYP107A1 interactions with 6-deoxyerythronolide. Amino acid residues within 5 Å of the substrate are shown in cyan. Hydrophobic residues are shown in orange. Water molecules are represented as red spheres, heme is green, and the substrate is blue. Amino acid residues are labelled according to their one-letter code. PDB code is within brackets next to the respective P450 name. A list of all amino acid residues shown in this figure is represented in Table 5.

CYP107A1, also known as P450eryF, catalyzes C6-hydroxylation of the macrolide 6-deoxyerythronolide B (6-DEB) in erythromycin biosynthesis [18]. CYP107A1 has an enlarged binding site pocket for the substrate, indicating it can accommodate larger molecules [18]. The substrate (6-DEB) is orientated perpendicular to the heme and interacts with three water molecules (Figure 2). Three phenylalanine residues lie within 5 Å of the substrate and may aid in sequestering and orientating the substrate (Figure 2) [18]. CYP107A1 activates molecular oxygen by cleaving the oxygen bond and inserting one oxygen atom into the C6 position of the substrate [18].

#### 3.3.2. Homotropic Cooperativity

**Figure 3:**
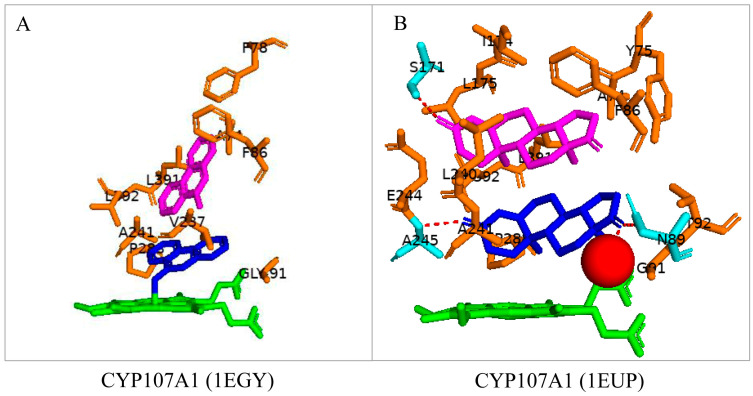
Homotropic cooperativity of CYP107A1 with 9-aminophenanthrene (**A**) and 4-androstene-3-17-dione (**B**). Amino acid residues within 5 Å of each ligand are shown. Active site residues that have polar interactions with the ligand are colored cyan, and those that have hydrophobic interactions are colored orange. Heme is shown in green; substrates are shown in blue and magenta. Water molecules are represented as red spheres. Amino acid residues are labelled according to their one-letter code. PDB code is within brackets next to the respective P450 name. A list of all amino acid residues shown in this figure is represented in Table 5.

Many P450s show homotropic cooperativity to various substrates, including polycyclic aromatic hydrocarbons and steroids [24]. This trend was also demonstrated with CYP107A1 when bound to 9-aminophenanthrene and 4-androstene-3-17-dione (Figure 3). Spectral binding analysis revealed the presence of two binding sites. The data indicated that the binding of the second ligand reduced the overall size and increased the hydrophobicity of the active site, which in turn increased the first ligand’s binding affinity [24]. Two molecules of each substrate were bound jointly within the binding pocket of CYP107A1. 9-aminophenanthrene interacted with various hydrophobic residues; one molecule was buried within the binding pocket and was in direct contact with the heme, whereas the second molecule was bound towards the surface of the pocket (Figure 3A) [24]. One molecule of 4-androstene-3-17-dione was found deep in the binding pocket, and instead of direct contact with the heme it interacted with a water molecule. It formed hydrogen bonds with active site residues, namely Asn-89 and Ala-245 (Figure 3B). The second molecule was towards the surface of the binding pocket. It showed a hydrogen bond with Ser-171 (Figure 3B)—various hydrophobic amino acid residues surrounded all the substrates, which could be involved in substrate anchoring. These results establish that homotropic cooperativity in ligand binding can arise because of the binding of two substrate molecules within the P450 active site [24].

#### 3.3.3. High Flexibility and Conformational Change of I-Helix Region

**Figure 4:**
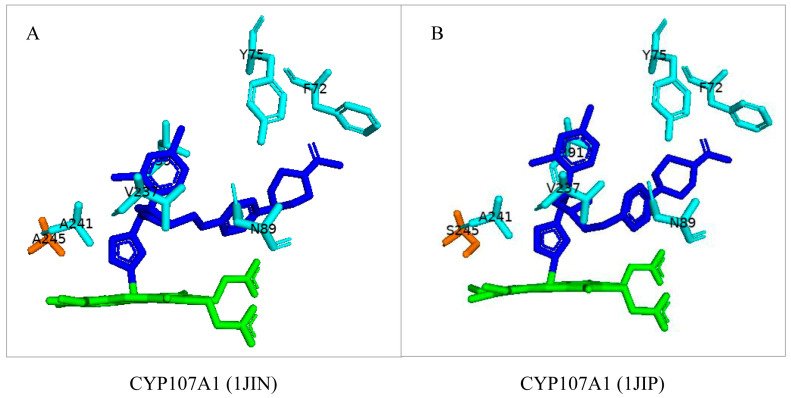
Analysis of interactions of native CYP107A1 (**A**) and mutant CYP107A1 (A245S) (**B**) bound with ketoconazole. Amino acids within 5 Å are shown in cyan. Substituted amino acid (Ala and Ser) is colored orange. Heme is shown in green, and substrate is shown in blue. Amino acid residues are labelled according to their one-letter code. PDB code is within brackets next to the respective P450 name. A list of all amino acid residues shown in this figure is represented in Table 5.

CYP107A1 can accommodate a large substrate, such as the azole P450 inhibitor ketoconazole, which is known to bind to many P450s [48]. Upon binding of this inhibitor, the I-helix of CYP107A1 was found to be unwound (Figure 4A) [25]. The flexibility of the I-helix cleft was also assessed by substituting the Ala-245 with a hydroxyl-containing residue, serine. Surprisingly, despite having a hydrogen-bonding amino acid (in the mutant), the I-helix was again found to unwind upon binding with ketoconazole (Figure 4B). However, when the natural substrate, 6-DEB, was added to the mutant, the I-helix reformed, revealing the flexibility of the active site of CYP107A1 [25].

#### 3.3.4. Ala-245 Is Critical to the Proton Shuttle

**Figure 5:**
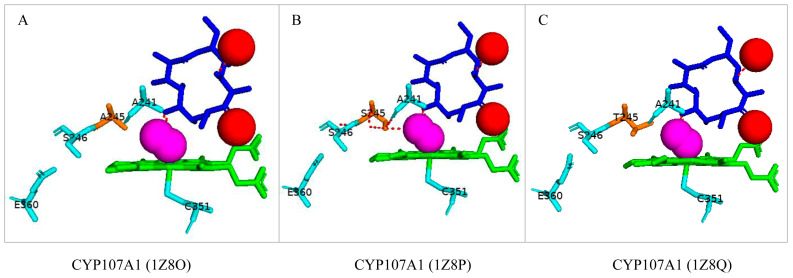
Analysis of the effect of Ala-245 substitution on the interactions of CYP107A1 with 6-deoxyerythronolide B (6-DEB). Interactions of 6-DEB with Native (**A**), A245S (**B**), and A245T (**C**) CYP107A1 are shown in the figure. Amino acids within 5 Å are shown in cyan. Substituted amino acid is colored orange. Dioxygen is shown as magenta, and water molecules as red spheres. Heme is shown in green, and substrate is shown in blue. Polar interactions are shown as red dashed lines. Amino acid residues are labelled according to their one-letter code. PDB code is displayed within brackets next to the respective P450 name. A list of all amino acid residues shown in this figure is represented in Table 5.

Thr-252 in the active site of numerous P450s has been proposed to participate in oxygen (O_2_) binding and its cleavage [49]. Interestingly, CYP107A1 has alanine instead of threonine [18,26]. The proton shuttle system in CYP107A1 was studied, and it was revealed that substituting Ala-245 to either serine or threonine led to an overall decrease in enzyme function. The substrate’s 5-OH is the only direct hydrogen bond donor to the iron-linked dioxygen and is probably a direct proton donor [26], as shown in Figure 5A. The wild-type CYP107A1 bound with 6-DEB and dioxygen revealed that one water molecule was expelled from the active site (Figure 5A). Introducing serine instead of alanine led to hydrogen bonding between the adjacent residue and the dioxygen molecule, breaking the proton shuttle pathway and decreasing enzyme activity (Figure 5B) [26]. The threonine mutant showed decreased enzyme activity as the hydroxyl group of threonine was too far away from the adjacent serine residue for any hydrogen bonds to form, disrupting the proton shuttling pathway (Figure 5C) [26]. These results show that Ala-245 plays a crucial role in CYP107A1, i.e., to increase the size of the active site cavity to accommodate larger substrates and for a functional proton shuttle system for the catalytic activity.

### 3.4. CYP107L1: Asp-50, Glu-85, and Glu-94 Play Pivotal Roles in the Substrate Access, Orientation, and Relocation within the Active Site

**Figure 6:**
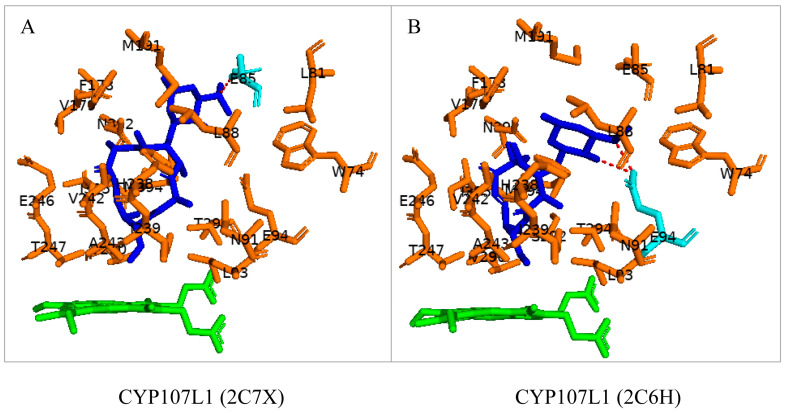
Substrate orientation within the active site of CYP107L1. (**A**) represents narbomycin and (**B**) represents YC-17. Amino acid residues sharing hydrophobic interactions are shown in orange. Amino acid residues with polar interactions are shown in cyan. Heme is shown in green, and substrates are shown in blue. Polar interactions are shown as red dashed lines. Amino acid residues are labelled according to their one-letter code. PDB code is displayed within brackets next to the respective P450 name. A list of all amino acid residues shown in this figure is represented in Table 5.

CYP107L1 is known for its substrate tolerance and for performing multiple hydroxylation’s on structurally diverse macrolides with the deoxyamino sugar desosamine [20,28]. Two of these steps include the hydroxylation of YC-17 at C10/C12 and at C12/C14 of narbomycin (Table 1) [20]. Interestingly, both substrates bind to the active site in similar orientations. However, the narbomycin deoxysugar C3′dimethylamino group forms a salt bridge with Glu-85 (Figure 6A), and YC-17 desosamine moiety C3′ forms a salt bridge with Glu-94 (Figure 6B). Interestingly, a comparative analysis of amino acid residues within the binding pocket of both open and closed conformations of CYP107L1 revealed that Glu-94 is present in the closed conformation only (Table 4). This indicates that CYP107L1 active site dynamics change when a substrate is bound within the binding pocket and incorporates Glu-94 to form a salt bridge with the substrate. Both substrates are surrounded by hydrophobic interactions with YC-17, securing the substrate further within the binding pocket.

**Figure 7:**
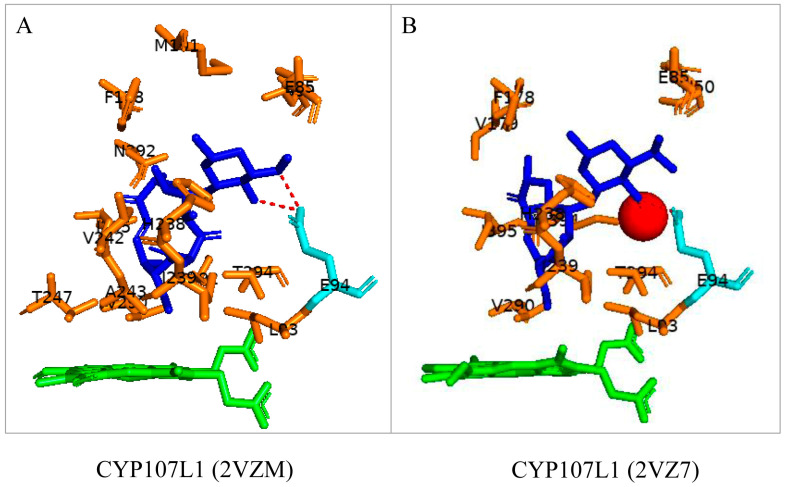
Analysis of substrate interactions with double mutants of CYP107L1. (**A**) represents D50N/E85Q mutant and narbomycin, and (**B**) represents D50N/E94Q and YC-17. Hydrophobic amino acid residues are shown in orange; polar interaction residues are shown in cyan. Polar interactions are shown as red dashed lines. Water molecules are shown as red spheres. Heme is shown as green, and substrates are shown as blue. Amino acid residues are labelled according to their one-letter code. PDB code is displayed within brackets next to the respective P450 name. A list of all amino acid residues shown in this figure is represented in Table 5.

To further investigate the importance of these negatively charged residues in substrate anchoring, these amino acid residues were substituted with neutral amino acid residues. Aspartic acid was substituted with asparagine, and glutamic acid was substituted with glutamine [28]. These site-directed mutants showed decreased catalytic function. These experiments proved the salt bridge formation is critical for substrate anchoring and catalytic function [20,28]. In these mutants, narbomycin was found deeper in the binding pocket, similar to YC-17 in the wild-type enzyme (Figure 7A). The orientation of YC-17 in the mutant and the wild-type were exactly the same (Figure 7B). Removing the negative charge of aspartic acid located near the entrance of the binding pocket resulted in narbomycin moving further into the binding pocket as the ionic bond formed in the wild-type was consequently removed [28]. This indicated a two-step substrate binding mechanism. The substrate first binds to the surface binding pocket and then moves to the buried binding pocket [28]. Asp-50 appears to function as a gate for substrate access, Glu-85 may play a role in substrate relocation, and Glu-94 plays a major role in substrate orientation for effective catalysis [28].

### 3.5. CYP107BR1: Distant Amino Acids Cause Conformational Change and Consequently Increase Enzymatic Activity

**Figure 8:**
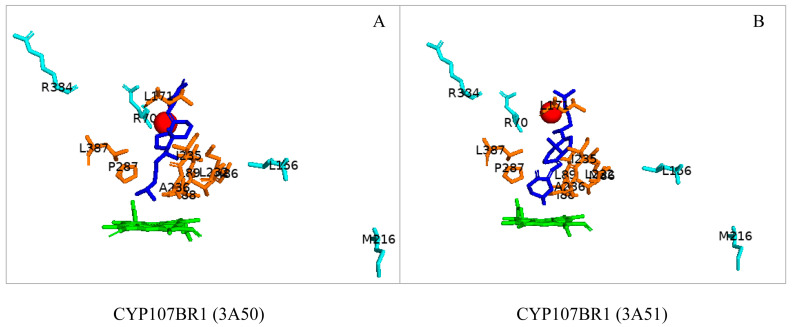
Analysis of substrate orientation in the active site cavity of a quadruple mutant of CYP107BR1 (Vdh-K1). (**A**) represents vitamin D3, and (**B**) represents 25(OH)VD3. Amino acid residues within 5 Å are shown in orange, and substituted amino acid residues are shown in cyan. Water molecules are represented as red spheres. Heme is shown in green, and substrates are shown in blue. Amino acid residues are labelled according to their one-letter code. PDB code is displayed within brackets next to the respective P450 name. A list of all amino acid residues shown in this figure is represented in Table 5.

CYP107BR1 performs a two-step hydroxylation reaction in the vitamin D3 synthesis pathway (Table 1) [23]. A quadruple mutant (Vdh-K1) of this P450 showed that random amino acid substitutions of four distant amino acids, namely T70R/V156L/E216M/E384R, that are not part of the binding pocket, resulted in increased enzyme activity (Figure 8) [23]. These substitutions led to conformational changes and shifted the P450 to a closed conformation, increasing the binding efficiency to its redox ferredoxin partner and, thus, the substrate [30]. The structure of CYP107BR1 bound with vitamin D3 revealed that the C25 position of the substrate is positioned very close to the heme iron; once C25 is hydroxylated, 25-hydroxyvitamin D3 is then bound to CYP107BR1, where the 3β-OH group is orientated close to the heme iron, which enables the 1α-hydroxylation step, as shown in Figure 8 [23]. The anti-parallel orientation indicates that this mutant is not functionally specialized. Ile-88 and Ile-150 were present in the closed conformation only (Table 4). Isoleucine is an aliphatic and hydrophobic amino acid that prefers to be buried in protein hydrophobic cores [50]. This amino acid, amongst others, is known to surround the aliphatic side chain and CD-rings of vitamin D3, thus explaining its addition to the closed conformation of this P450 (Figure 8) [23].

### 3.6. CYP107E1: Orientation of Substrate Sugar Moieties Determines Catalytic Function

**Figure 9:**
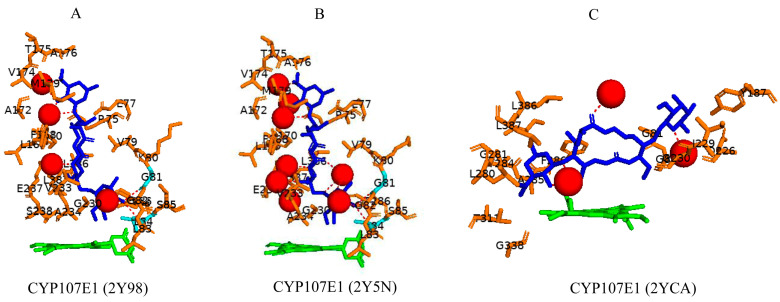
Substrate orientation within the active site of CYP107E1. (**A**) represents mycinamicin IV, (**B**) represents mycinamicin V, and (**C**) represents mycinamicin III. Amino acid residues within 5 Å are shown in orange, and amino acid residues sharing a polar interaction with the substrate are shown in cyan. Water molecules are represented as red spheres. Heme is shown in green, and substrates are shown in blue. Polar interactions are shown as red dashed lines. Amino acid residues are labelled according to their one-letter code. PDB code is displayed within brackets next to the respective P450 name. A list of all amino acid residues shown in this figure is represented in Table 5.

CYP107E1 is a multi-functional P450 in the mycinamycin synthesis pathway, performing sequential hydroxylation and epoxidation reactions (Table 1) [19]. Mycinamycin IV (M-IV) and mycinamycin V (M-V) are native substrates of this P450 as opposed to mycinamycin III (M-III), which is the biosynthetic precursor [19]. M-III contains the monomethoxy sugar javose instead of dimethoxylated mycinose. Both M-IV and M-V are bound orthogonally to the heme, following a pattern of “mycinose-in and desosamine-out” [19], and the C-12-C14 was near the heme iron (Figure 9A,B). Gly-81, Leu-84, Gly-230, and Val-233 are part of the active site cavity of CYP107E1 in the closed conformation only (Table 4). Gly-81 and Leu-84 form hydrogen bonds with mycinamycin IV (Figure 9A), and Gly-81, Leu-84, and Val-233 formed hydrogen bonds with the substrate, mycinamycin V (Figure 9B). These substrates are known to have multiple interactions with the BC loop, FC loop, and I-helix of the P450 structure. Gly-81 and Leu-84 are found within the BC loop, and Val-233 is located in the I-helix, thus explaining the hydrogen bond interactions [19]. M-III is bound parallel to the heme plane following a pattern of “desosamine-in and javose-out” [19] and is buried more deeply in the binding pocket. However, consistent with the poor functional turnover of M-III, the reactive centers C-14 and C12–C13 are not exposed to the heme iron (Figure 9C). This indicates that javose is less preferable than mycinose as an initial recognition marker. For the substrate to reach a catalytically productive mode, mycinose instead of desosamine should lead the way [19].

### 3.7. CYP107G1: Ala-241 and Gly-242 Are Involved in Substrate Anchoring

**Figure 10:**
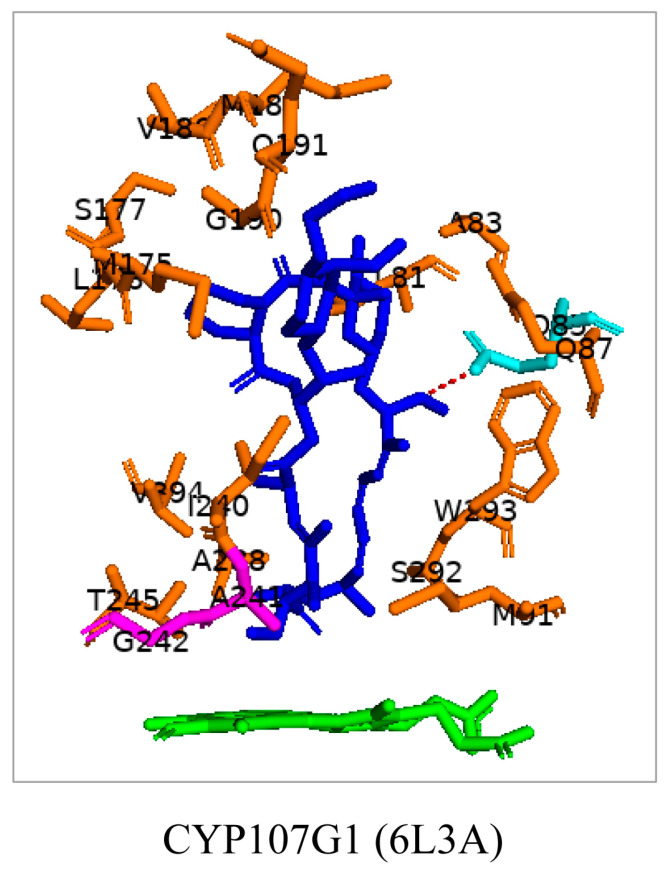
Analysis of interaction of CYP107G1 with everolimus. Residues within 5 Å are shown in orange. Amino acid residues sharing a polar interaction with the substrate are shown in cyan. Amino acid residues involved in substrate anchoring are shown in magenta. The heme is shown in green, and the substrate is shown in blue. Amino acid residues are labelled according to their one-letter code. The polar interaction between substrate and amino acid residue is shown with red dashed lines. PDB code is displayed within brackets next to the respective P450 name. A list of all amino acid residues shown in this figure is represented in Table 5.

CYP107G1 can catalyze the specific oxidation C-27 in the pre-rapamycin macrolide molecule (Table 1) [21]. Everolimus is a clinical derivative of rapamycin; this substrate is bound compressed and perpendicular to the heme (Figure 10). Ala-241 and Gly-242 were found to be involved in anchoring the substrate to ensure the correct orientation for the reaction [21]. Leu-109 and Ile-154 were present in the closed conformation of this enzyme (Table 4). These hydrophobic residues may aid in anchoring the substrate once bound.

### 3.8. CYP107E6: Arg-81 and Arg-190 Play a Role in Substrate Orientation

**Figure 11:**
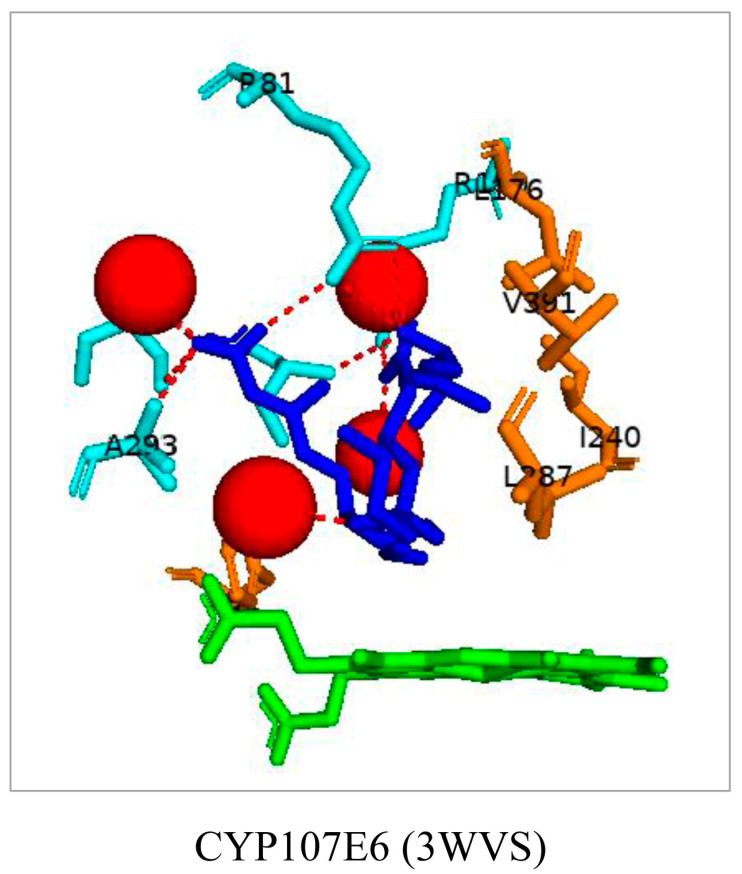
Analysis of CYP107E6 interactions with reveromycin T. Amino acid residues sharing a hydrophobic interaction are shown in orange. Amino acid residues sharing a polar interaction with the substrate are shown in cyan. Water molecules are represented as red spheres. The heme is shown in green, and the substrate is shown in blue. Polar interactions are shown as red dashed lines. Amino acid residues are labelled according to their one-letter code. PDB code is displayed within brackets next to the respective P450 name. A list of all amino acid residues shown in this figure is represented in Table 5.

CYP107E6, commonly known as P450revI, catalyzes the C18-hydroxylation of reveromycin T in the biosynthetic pathway of reveromycin A (RM-A), a promising lead compound with anti-osteoclastic activity (Table 1) [34]. The substrate binds to the active site where Arg-190 forms a salt bridge with the C1 carboxyl group, and Arg-81, which is located at the entrance of the binding pocket, forms a bifurcated hydrogen bond with the C5 hydroxyl and C24 carboxyl groups of the substrate (Figure 11) [34]. The oxygen atom of reveromycin T forms a water-mediated hydrogen bond (Figure 11) [34]. These interactions enable the correct substrate orientation, where C18 is positioned near the heme iron ready for catalysis to occur.

### 3.9. CYP107FH5: Steric and Hydrophobic Interactions with Phe-92, Leu-399, and Ile-400 Are Critical for the C-10 Hydroxylation Step in the Tirandamycin Pathway

CYP107FH5, commonly known as TamI, is a multi-functional P450 involved in the hydroxylation and epoxidation steps in the tirandamycin biosynthetic pathway (Table 1) [36]. This P450 performs a C10 hydroxylation, converting tirandamycin C to tirandamycin E, followed by a C11/C12 epoxidation, converting tirandamycin D to tirandamycin A, and finally, a C18 hydroxylation, converting tirandamycin A to tirandamycin B (Table 1) [36]. The second step of the pathway, i.e., conversion of tirandamycin E to tirandamycin D, is performed by the FAD-dependent oxidase, TamL [36].

**Figure 12:**
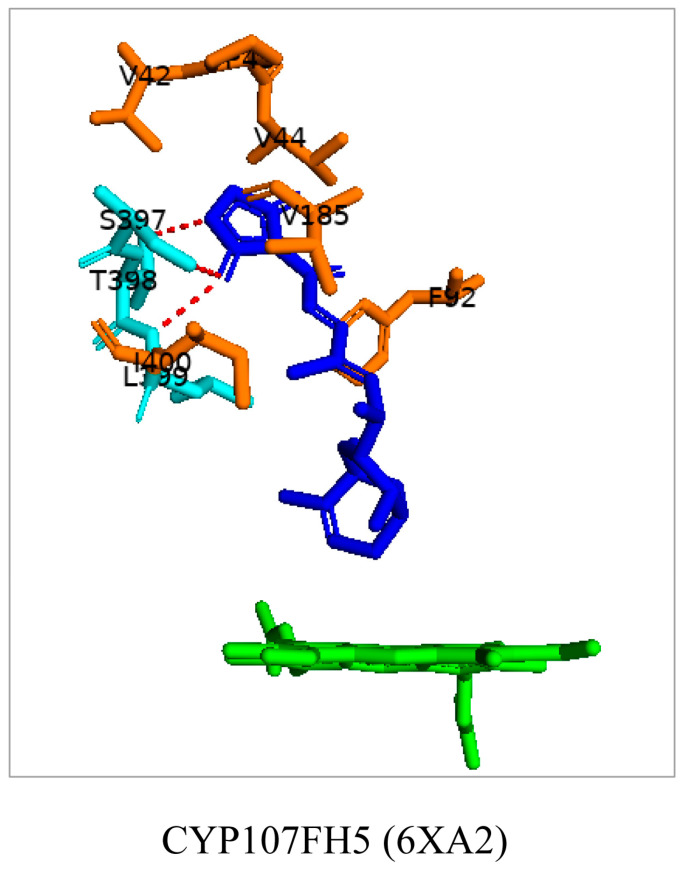
Analysis of CYP107FH5 interactions with tirandamycin C. Amino acid residues sharing a hydrophobic interaction are shown in orange. Amino acid residues sharing a polar interaction with the substrate are shown in cyan. Heme is shown in green, and substrate is shown in blue. Polar interactions are shown as red dashed lines. Amino acid residues are labelled according to their one-letter code. PDB code is displayed within brackets next to the respective P450 name. A list of all amino acid residues shown in this figure is represented in Table 5.

CYP107FH5 performs the initial hydroxylation step on tirandamycin C to produce tirandamycin E. This substrate comfortably binds within the active site pocket with the oxidation site near the heme, surrounded by hydrophobic residues such as Val-42, Pro-43, Val-44, Phe-92, Val-185, and Ile-400 (Figure 12). Ser-397, Thr-398, and Leu-399 form hydrogen bonds with the substrate (Figure 12). Interestingly, substituting Ser-397 and Thr-398 resulted in no change in the substrate recognition or binding, proving that these polar interactions with the substrate were not responsible for substrate anchoring [36]. However, the mutation of Leu-399 along with Phe-92 and Ile-400, which are near the substrate’s polyene chain, resulted in decreased or complete loss of enzymatic activity due to a disturbance in substrate binding [36]. These data indicated that non-polar and steric interactions with the aromatic and hydrophobic residues Leu-399, Ile-400, and Phe-92 are required for the correct orientation of the substrate in relation to the heme-iron and are thus essential for the C-10 hydroxylation reaction to occur [36].

## 4. Conclusions

Understanding the structure–function relationship between a protein and its ligands is critical in designing novel enzymes with potential biotechnological applications. The availability of numerous protein crystal structures with many bound ligands is ideal for achieving this goal. The present study attempts to apply this strategy to the P450 superfamily, focusing on multifunctional and catalytically diverse P450 family CYP107.

Members of the CYP107 family are well-known for their capacity to perform oxidation and epoxidation reactions on various substrates of pharmaceutical and biotechnological importance. These multifunctional P450s are typically integrated into natural metabolite biosynthetic gene clusters, particularly in macrolide antibiotic gene clusters, and undertake oxidative tailoring processes to increase efficacy or attribute structural diversity to natural metabolites. The CYP107 proteins studied typically have a large and flexible active site to handle larger and multiple substrates. Structure–function analysis revealed that the size of the substrate carbon skeleton can eject water molecules from the active site, potentially making room for a larger substrate. Additionally, the type of sugar moiety attached to the substrate backbone determines the correct orientation within the binding pocket and impacts catalytic performance. Furthermore, homotropic cooperativity for steroids and PAH and I-helix flexibility is observed for CYP107 enzymes. Amino acids within the binding pocket are shown to be involved in substrate anchoring and orientation. Finally, some residues formed critical salt bridges or hydrophobic interactions, or were engaged in proton shuttle systems in specific CYP107s to ensure that catalysis could occur. These polar and hydrophobic interactions forced the substrate to remain in a specific location within the binding pocket. They ensured that the oxidation site of the substrate was placed near the heme iron for the catalytic reaction to proceed. Amino acid dynamics and site-directed mutagenesis work revealed critical amino acid substitutions that interacted with the substrate.

Interestingly, research on CYP107BR1 found that distant amino acid residues not located within the substrate binding region may also alter P450 function. To our knowledge, CYP107A1 is still the only P450 in the 107 family that lacks the conserved threonine residue. Instead, it has an alanine residue, which has been proven critical in the function of CYP107A1. CYP107 members were found to be highly flexible concerning their adaptable and large active site that allows access to various substrates. When various-sized substrates were bound, the hydrophobic regions inside the active site changed to accommodate the particular substrate, demonstrating their flexibility and tendency to interact with it. RMSD values between open and closed conformations revealed that the amino acid residues considerably shifted when an elongated substrate was bound within the active site. Its dynamic behavior is comparable to well-studied P450s such as CYP102 and CYP109.

This study provides comprehensive information on CYP107 family members’ structure–function analysis and highlights the active site cavity dynamics and amino acids’ role in catalysis. This work will act as a further guide to future genetic engineering of CYP107 enzymes to produce novel molecules of medical and biotechnological interest.

## Figures and Tables

**Table 1:** Role of CYP107s in the synthesis of primary and secondary metabolites. Key reactions are shown, and metabolite(s) reaction sites are highlighted in red.

P450	Species Name	Biological Process and Enzymatic Reaction	The Biological Significance of the Product	Reference
CYP107H1 (P450BioI)	*Bacillus subtilis*	Pimelic acid synthesis C7–C8 carbon–carbon bond cleavage 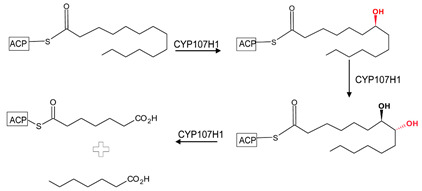	Vitamin	[22]
CYP107A1 (P450eryF)	*Saccharopolyspora erythraea* NRRL23338	Erythromycin biosynthesis C6-hydroxylation of the macrolide 6-deoxyerythronolide B (6-DEB) 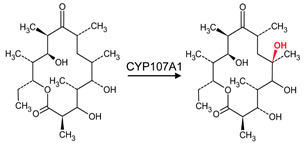	Antibacterial agent	[18,24,25,26]
CYP107U1	*Streptomyces coelicolor* A3(2)	Glycocholic acid biosynthesis Dehydrogenation of glycocholic acid to glyco-7-oxo-deoxycholic acid 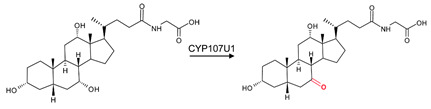	Detergent	[27]
CYP107L1 (PikC)	*Streptomyces venezuelae*	Pikromycin biosynthesis Mono-hydroxylation at C12 of the 12-membered ring macrolactone of YC-17 to produce methymycin 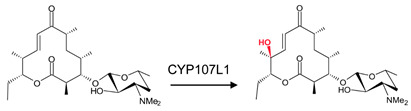 Mono-hydroxylation at C10 of the 12-membered ring macrolactone of YC-17 to produce neomethymycin 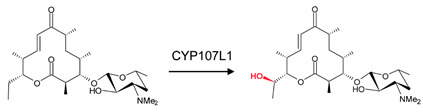 Dihydroxylation of YC-17 results in novamethymycin 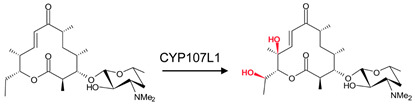 Hydroxylation of C12 of the 14-membered ring of narbomycin, giving rise to pikromycin 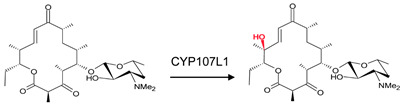 Hydroxylation at the C14 position of narbomycin, giving rise to neopikromycin 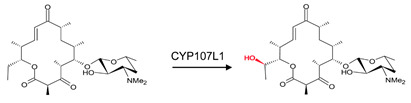 Dihydroxylation of narbomycin to yield novapikromycin 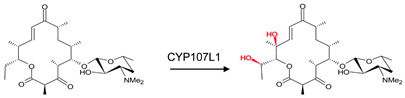	Antibacterial agents	[20,28]
CYP107BR1 (P450vdh)	*Pseudonocardia autotrophica*	Vitamin D biosynthesis Two-step hydroxylation of VD3; these reactions are equivalent to those catalyzed by the human enzymes CYP27A1 (also CYP2R1) and then CYP27B1 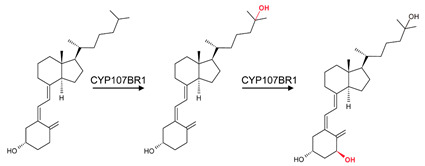	Vitamin	[23,29,30]
CYP107E1 (MycG)	*Micromonospora griseorubida*	Mycinamicin biosynthesis Sequential hydroxylation and epoxidation reactions at two distinct sites, a tertiary allylic C–H bond (C-14) and an olefin (C12–C13) C14-Hydroxylation and C12/C13-epoxidation on macrolactone ring of mycinamicin 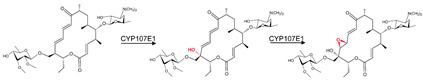	Antibacterial agent	[19]
CYP107B (HmtT)	*Streptomyces himastatinicus* ATCC 53653	Himastatin biosynthesis Catalyzes the formation of hexahydropyrroloindole from L-tryptophan in the himastatin synthesis pathway 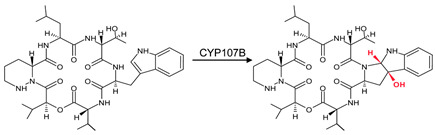	Antibacterial agent	[31]
CYP107C1 (orfA)	*Streptomyces thermotolerans*	Carbomycin biosynthesis C12–C13 epoxidation of carbomycin B to make carbomycin A 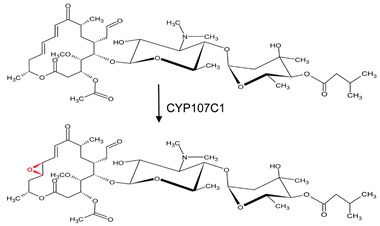	Antibacterial agent	[32,33]
CYP107G1 (rapN)	*Streptomyces hygroscopicus*	Rapamycin biosynthesis Specific oxidation of C-27 in the pre-rapamycin macrolide molecule 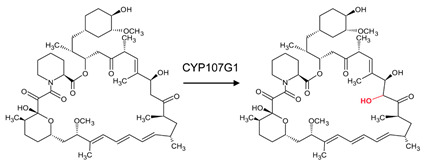	Immunosuppressive, antifungal, and antitumor agent	[21]
CYP107E6 (P450revI)	*Streptomyces* sp. SN-593	Reveromycin T biosynthesis C18-hydroxylation of Reveromycin T 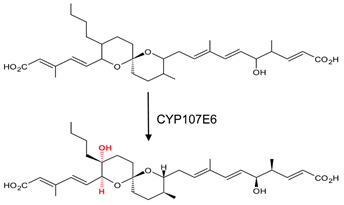	Anabolic agent for the treatment of osteoporosis.	[34]
CYP107W1	*Streptomyces avermitilis*	Oligomycin biosynthesis C12-hydroxylation reaction of oligomycin C to form oligomycin A 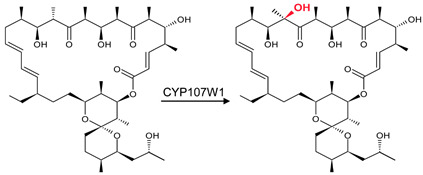	Antibiotic	[35]
CYP107FH5 (CYP TamI)	*Streptomyces* sp. 307-9	Tirandamycin biosynthesis C10 hydroxylation, oxidative conversion of C10 hydroxyl to carbonyl, C11/C12 epoxidation, C18 hydroxylation 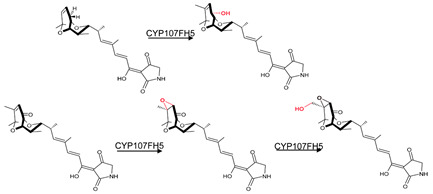	Antibiotic	[36]
CYP107Z14 (P450-sb21)	*Sebekia benihana*	Cyclosporine A pathway Hydroxylating at the 4th *N*-methyl leucine (MeLeu4) 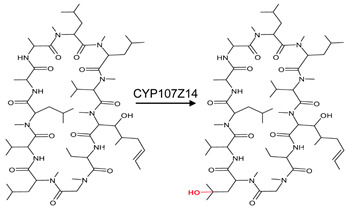	Immunosuppressant	[37]
CYP107X1	*Streptomyces avermitilis*	Progesterone pathway The 16α-Hydroxylation of progesterone 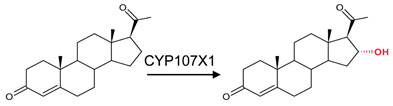	Steroid	[38]

**Table 2:** CYP107 crystal structures used in the study. The PDB codes, P450 names, and their references are presented.

PDB Code	P450 Name	Reference
3EJB, 3EJD, 3EJE	CYP107H1	[22]
1JIO, 1JIN, 1JIP, 1Z8O, 1Z8P, 1Z8Q, 1OXA, 1EGY, 1EUP	CYP107A1	[18,24,25,26]
2BVJ, 2CD8, 2C7X, 2C6H, 2VZ7, 2VZM, 2WHW, 2WI9	CYP107L1	[20,28]
3A4G, 3A50, 3A4H, 3A4Z, 3A51, 3VRM, 5GNM, 5GNL	CYP107BR1	[23,29,30]
2YGX, 2YCA, 2Y98, 2Y5Z, 2Y5N, 3ZSN, 4AW3	CYP107E1	[19]
4GGV	CYP107B	[31]
6L39, 6L3A	CYP107G1	[21]
7WEX	CYP107X1	[38]
4WPZ	CYP107W1	[35]
6XA2, 6XA3	CYP107FH5 *	[36]
6M4S	CYP107Z14 *	[37]
3WVS	CYP107E6 *	[34]

* P450s named in this study.

**Table 3:** The 41 CYP107 crystal structures’ active site cavity area, volume, and conformations.

P450 Name	PDB Code	Area (SA) Å^2^	Volume (SA) Å^3^	Conformation
CYP107A1	1EGY	848	701	Closed
CYP107A1	1EUP	851	751	Closed
CYP107A1	1Z8Q	824	647	Closed
CYP107A1	1JIP	903	729	Closed
CYP107A1	1JIN	855	691	Closed
CYP107A1	1Z8P	816	655	Closed
CYP107A1	1JIO	841	700	Closed
CYP107A1	1Z8O	829	664	Closed
CYP107A1	1OXA	837	715	Closed
CYP107BR1	3VRM	1098	970	Closed
CYP107BR1	3A50	1231	1066	Closed
CYP107BR1	3A51	1260	1064	Closed
CYP107E1	2Y5N	1600	1189	Closed
CYP107E1	2Y98	1459	994	Closed
CYP107E1	2Y5Z	1552	1118	Closed
CYP107E1	3ZSN	1520	1286	Closed
CYP107E1	4AW3	1623	1412	Closed
CYP107G1	6L3A	2070	2196	Closed
CYP107L1	2C6H	1180	933	Closed
CYP107L1	2C7X	1124	711	Closed
CYP107L1	2CD8	1148	793	Closed
CYP107L1	2VZ7	1131	934	Closed
CYP107L1	2VZM	1181	943	Closed
CYP107L1	2WHW	1171	921	Closed
CYP107L1	2WI9	1177	922	Closed
CYP107E6	3WVS	1542	1121	Closed
CYP107FH5	6XA2	789	455	Closed
CYP107E1	2YCA	1490	1261	Closed
CYP107B	4GGV	1369	954	Open
CYP107BR1	3A4G	1448	1659	Open
CYP107BR1	3A4H	1333	1536	Open
CYP107BR1	5GNM	1294	1426	Open
CYP107BR1	5GNL	1271	1447	Open
CYP107E1	2YGX	1526	1033	Open
CYP107G1	6L39	2287	2637	Open
CYP107L1	2BVJ	1151	871	Open
CYP107W1	4WPZ	2021	1943	Open
CYP107X1	7WEX	1720	1520	Open
CYP107FH5	6XA3	1263	1763	Open
CYP107Z14	6M4S	950	859	Open
CYP107BR1	3A4Z	1249	1093	Open

**Table 4:** Analysis of amino acid dynamics for CYP107 subfamilies. The unique amino acid residues found within 5 Å of the heme in the active site cavities of five CYP107 proteins belonging to the same subfamily in open and closed conformations are presented in the table.

P450 Name	Conformation	Number of Amino Acids in the Active Site Cavity	Unique Amino Acids	Amino Acids Interacting with the Substrate	RMSD (Å)
CYP107BR1	Open	35	Leu153	-	2.5
	Closed	34	Ile88 and Ile150	Ile88	
CYP107L1	Open	36	Leu93, His245, Val290, Thr294	-	0.6
	Closed	36	Glu94, Val291, Thr295, Ala357	Glu94	
CYP107E1	Open	35	Ala285	-	0.4
	Closed	33	Gly81, Leu84, Gly230, Val233	Gly81, Leu84, Val233	
CYP107G1	Open	31	Leu282	-	0.7
	Closed	30	Leu190, Ile154	Gln85	
CYP107FH5	Open	35	Thr70, Leu166	-	3.0
	Closed	35	Ile163, Ala362	Ser397, Thr398, Leu399	

**Table 5:** List of amino acid residues within 5 Å of the ligand of CYP107 proteins. Amino acid residues share a polar interaction with the ligand, shown in bold.

CYP Name (PDB Code)	Amino Acid Residues
CYP107H1 (3EJB)	Lys35, **Tyr36**, Pro37, Ser56, **Arg59**, Thr60, Pro61, Leu62, Pro63, Glu64, Gln76, Met79, Leu81, Phe82, Leu164, Ile165, Thr167, **Ile168**, Phe170, **Arg172**, Leu230, Ile233, Ala234, Thr238, Thr281, Met283, Thr284, Ala285, Gln304, **Tyr306**, Phe383
CYP107H1 (3EJD)	Lys35, Tyr36, Pro37, Ser56, **Arg59**, Thr60, **Pro61**, **Leu62**, Pro63, Glu64, Gln76, Met79, Leu81, Phe82, Leu164, Ile165, Thr167, **Ile168**, Phe170, **Arg172**, Leu230, Ile233, Ala234, Thr238, Thr281, Met283, Thr284, Ala285, Gln304, Tyr306, Phe383
CYP107H1 (3EJE)	Lys35, Tyr36, Pro37, Ser56, **Arg59**, Thr60, Pro61, Leu62, Pro63, Glu64, Gln76, Met79, Leu81, Phe82, Leu164, Ile165, Thr167, **Ile168**, Phe170, **Arg172**, Leu230, Ile233, Ala234, Thr238, Thr281, **Met283**, Thr284, Ala285, Gln304, **Tyr306**, Phe383
CYP107A1 (1OXA)	**Ala74**, **Tyr75**, Phe78, **Phe86**, **Asn89**, Gly91, **Thr92**, Ser171, **Ile174**, **Leu175**, Arg185, **Val237**, Leu240, **Ala241**, Phe243, Glu244, Ala245, Phe288, Leu390, **Leu391**
CYP107A1 (1EGY)	Ala74, Tyr75, Phe78, Phe86, Asn89, Gly91, Thr92, Ile174, Leu175, Arg185, Val237, Ala241, Phe243, Ala245, Pro288, Leu391, Leu392
CYP107A1 (1EUP)	Ala74, Tyr75, Phe78, Phe86, Ala87, **Asn89**, Met90, Gly91, Thr92, **Ser171**, Ser172, Ile174, Leu175, Arg185, Leu236, Val237, Leu240, Ala241, Phe243, Glu244, **Ala245**, Pro288, Leu391, Leu392
CYP107A1 (1JIN)	Ser58, Ser59, Asp60, Pro61, Phe72, Ala74, Tyr75, Asn89, Gly91, Thr92, Ile174, Leu175, Arg185, Val237, Leu238, Leu240, Ala241, Glu244, Ala245, Thr291, Thr292, Arg293, Phe294, Leu391, Leu392
CYP107A1 (1JIP)	Ser58, Ser59, Asp60, Pro61, Phe72, Ala74, Tyr75, Asn89, Gly91, Thr92, Ile174, Leu175, Arg185, Val237, Leu238, Leu240, Ala241, Glu244, Ser245, Thr291, Thr292, Arg293, Phe294, Leu391, Leu392
CYP107A1 (1Z8O)	Ala74, Tyr75, Phe78, Phe86, Asn89, Gly91, Thr92, Ser171, Ile174, Leu175, Val237, Leu240, Ala241, Glu244, Ala245, Ser246, Pro288, Thr291, Cys351, Glu360, Leu391, Leu392
CYP107A1 (1Z8P)	Ala74, Tyr75, Phe78, Phe86, Asn89, Gly91, Thr92, Ser171, Ile174, Leu175, Val237, Leu240, Ala241, Glu244, Ser245, Ser246, Pro288, Thr291, Cys351, Glu360, Leu391, Leu392
CYP107A1 (1Z8Q)	Ala74, Tyr75, Phe78, Phe86, Asn89, Gly91, Thr92, Ser171, Ile174, Leu175, Val237, Leu240, Ala241, Glu244, Thr245, Ser246, Pro288, Thr291, Cys351, Glu360, Leu391, Leu392
CYP107L1 (2C7X)	Asp50, Trp74, **Glu85**, Leu81, Leu83, Leu88, Asn91, Leu93, Glu94, Phe178, Val179, Ala187, Gln188, Met191, His238, Ile239, Val242, Ala243, Glu246, Thr247, Val290, Thr294, Tyr295, Asn392, Met394, Ile395
CYP107L1 (2C6H)	Asp50, Trp74, Glu85, Leu81, Leu83, Leu88, Asn91, Leu93, **Glu94**, Phe178, Val179, Ala187, Gln188, Met191, His238, Ile239, Val242, Ala243, Glu246, Thr247, Val290, Thr294, Tyr295, Asn392, Met394, Ile395
CYP107L1 (2VZM)	Asp50, Trp74, Glu85, Leu81, Leu83, Leu88, Asn91, Leu93, **Glu94**, Phe178, Val179, Ala187, Gln188, Met191, His238, Ile239, Val242, Ala243, Glu246, Thr247, Val290, Thr294, Tyr295, Asn392, Met394, Ile395
CYP107L1 (2VZ7)	Asp50, Trp74, Glu85, Leu81, Leu83, Leu88, Asn91, Leu93, **Glu94**, Phe178, Val179, Ala187, Gln188, Met191, His238, Ile239, Val242, Ala243, Glu246, Thr247, Val290, Thr294, Tyr295, Asn392, Met394, Ile395
CYP107BR1 (3A50)	Trp67, Pro83, Thr84, Met86, Ile88, Leu89, Leu171, Val172, Ala177, Lys180, Asn181, Met184, Leu232, Ile235, Ala236, Thr240, Val283, Pro287, Leu387
CYP107BR1 (3A51)	Trp67, Pro83, Thr84, Met86, Ile88, Leu89, Leu171, Val172, Ala177, Lys180, Asn181, Met184, Leu232, Ile235, Ala236, Thr240, Val283, Pro287, Leu387
CYP107E1 (2Y98)	Arg75, Glu77, Val79, Lys80, **Gly81**, Gly82, Leu83, **Leu84**, Ser85, Phe168, Leu169, Ser170, Ala172, Val174, Thr175, Ala176, Glu178, Met179, Ala183, Gly230, Val233, Ala234, Glu237, Ser238, Phe286, Leu386, Leu387
CYP107E1 (2Y5N)	Arg75, Glu77, Met78, Val79, Lys80, **Gly81**, Gly82, Leu83, **Leu84**, Ser85, Phe168, Leu169, Ser170, Ala172, Val174, Thr175, Ala176, Glu178, Met179, Ala183, Gly230, Val233, Ala234, Glu237, Ser238, Phe286, Leu386, Leu387
CYP107E1 (2YCA)	Val79, Lys80, Gly82, Leu83, leu84, Phe168, Tyr187, Asp226, Ile229, Gly230, Val233, Ala234, Glu237, Ser238, Leu280, Gly281, Val282, Gly283, Thr284, Ala285, Phe286, Thr311, Gly338, Leu386, Leu387
CYP107G1 (6L3A)	Leu81, Ala83, **Gln85**, Gln87, Met91, Met175, Leu176, Ser177, val186, Met187, Gly190, Gln191, Ile240, Ala241, Gly242, Thr245, Ile287, Ala282, Ser292, Trp293, Val394
CYP107E6 (3WVS)	**Arg81**, Ala85, Ala87, Ser89, Phe91, Ile175, Leu176, **Arg190**, Asp233, Ile236, Gly237, Leu238, Ile240, Ala241, Thr245, Leu287, Gly288, Gly290, Ser291, Ala292, **Ala293**, Pro294, Leu318, Met390, Val391
CYP107FH5 (6XA2)	Val42, Pro43, Val44, Cys45, Ala91, Phe92, Leu101, His102, Leu184, Val185, Leu244, Ile247, Gly248, Glu251, Thr252, Leu295, His297, Ala298, Thr299, **Ser397**, **Thr398**, **Leu399**, Ile400

## Data Availability

Data are contained within the article.
